# EEG can Track the Time Course of Successful Reference Resolution in Small Visual Worlds

**DOI:** 10.3389/fpsyg.2015.01787

**Published:** 2015-11-20

**Authors:** Christian Brodbeck, Laura Gwilliams, Liina Pylkkänen

**Affiliations:** ^1^Department of Psychology, New York University, New York, NY, USA; ^2^Department of Linguistics, New York University, New York, NY, USA; ^3^NYUAD Institute, New York University Abu Dhabi, Abu Dhabi, United Arab Emirates

**Keywords:** EEG/ERP, reference resolution, visual short-term memory, contralateral activity, language comprehension, reading

## Abstract

Previous research has shown that language comprehenders resolve reference quickly and incrementally, but not much is known about the neural processes and representations that are involved. Studies of visual short-term memory suggest that access to the representation of an item from a previously seen display is associated with a negative evoked potential at posterior electrodes contralateral to the spatial location of that item in the display. In this paper we demonstrate that resolving the reference of a noun phrase in a recently seen visual display is associated with an event-related potential that is analogous to this effect. Our design was adapted from the visual world paradigm: in each trial, participants saw a display containing three simple objects, followed by a question about the objects, such as *Was the pink fish next to a boat?*, presented word by word. Questions differed in whether the color adjective allowed the reader to identify the referent of the noun phrase or not (i.e., whether one or more objects of the named color were present). Consistent with our hypothesis, we observed that reference resolution by the adjective was associated with a negative evoked potential at posterior electrodes contralateral to spatial location of the referent, starting approximately 333 ms after the onset of the adjective. The fact that the laterality of the effect depended upon the location of the referent within the display suggests that reference resolution in visual domains involves, at some level, a modality-specific representation. In addition, the effect gives us an estimate of the time course of processing from perception of the written word to the point at which its meaning is brought into correspondence with the referential domain.

## Introduction

Identifying the entities that individual expressions refer to is a fundamental prerequisite for understanding language in context. Even though EEG has been used widely to study language comprehension, so far no neural marker of successful reference resolution has been described. In this study we demonstrate that EEG can be used to track reference resolution by using visual displays as the referential domain. In this context, successful reference resolution is associated with an evoked potential known from research on visual short-term memory.

The cognitive basis of referential processing has been extensively studied with the so-called visual world paradigm (for a recent review, see Huettig et al., 2011). In these studies, participants typically look at a visual display while listening to instructions involving the display, and an eye tracker is used to determine what objects participants look at as the sentence unfolds. Results from visual world studies have underlined the centrality of referential processing for language comprehension by suggesting that the referential context can influence early syntactic parsing decisions (Tanenhaus et al., 1995; Spivey et al., 2002).

Concerning the process of reference resolution itself, visual world studies have suggested that it is fast and uses new information incrementally. When listeners followed spoken instructions such as “touch the starred yellow square,” they fixated the referent shortly after hearing the word that allowed them to uniquely identify the referent, i.e., in an environment with only one starred item they fixated that item shortly after the word “starred,” whereas in an environment with two starred items but only one of them yellow they fixated that item shortly after hearing “yellow” (Eberhard et al., 1995; Sedivy et al., 1999). Studies with more complex contexts have shown that eye movements in scenes are not just reactive to linguistic input but instead reflect listeners’ predictions about upcoming referents, by, for example, fixating on a cake when hearing the verb “eat” (Altmann and Kamide, 1999, 2007; Kamide et al., 2003).

While eye tracking studies with the visual world paradigm have shed light on various aspects of reference resolution, not much is known about the time course of corresponding neural processes. Indirect evidence comes from a group of EEG studies which established that referential ambiguity is associated with a sustained negative evoked potential at frontal electrode sites, identified as “Nref” (reviewed by Nieuwland and Van Berkum, 2008). With serial visual presentation, referentially ambiguous determiner-noun phrases evoked an Nref around 300 ms after presentation of the noun (Van Berkum et al., 1999). A similar effect to referentially ambiguous pronouns had an onset around 400 ms (Nieuwland and Van Berkum, 2006). These results establish a time frame for referential processing by showing when the brain starts responding to referential ambiguity. There is some evidence that the Nref is specific to referential ambiguity, as pronoun resolution difficulty from sources other than referential ambiguity is not associated with an Nref (Van Berkum et al., 2007). Another relevant EEG study used a continuously presented visual world and auditory sentence stimuli, reporting a late central positive “P600” effect, commonly associated with syntactic violations and ensuing reanalysis, in a 500–800 ms time window when it became clear based on the visual world that a grammatically acceptable language fragment had to be reanalyzed as a less preferred construction (Knoeferle et al., 2008). While not directly reflecting referential processing, this still demonstrates an interaction between visual and linguistic information.

**FIGURE 1:**
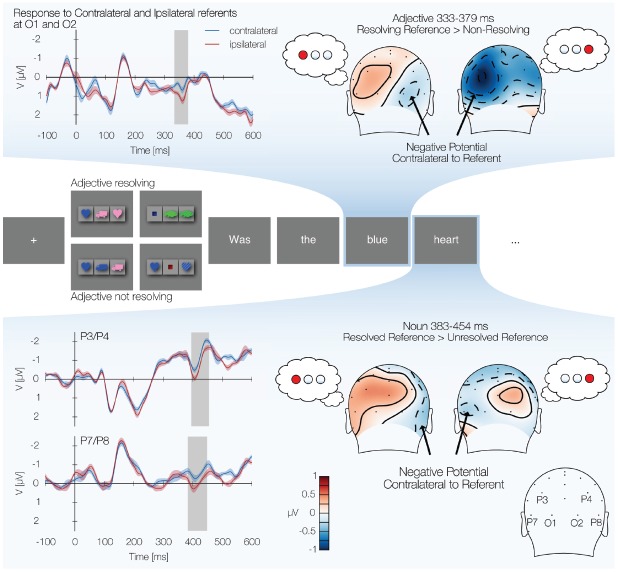
**Posterior negativity contralateral to the referents of adjectives and nouns. Top panel:** the left side shows the response to the adjective at electrodes O1 and O2, with responses grouped according to whether reference was resolved to the side contralateral or ipsilateral to the sensor. The shaded area indicates the region in which the response to contralateral referents was significantly more negative than the response to ipsilateral referents. The topographies on the right show difference maps of the average voltage during the time window established by the cluster, seen from the back of the head; for referents on the left and right, the average potential is plotted for resolving adjectives to that side minus all non-resolving adjectives. **Middle panel:** illustration of the paradigm, each rectangle representing a computer screen. Each trial started with a fixation cross. Next a visual world display was presented (the four different displays shown illustrate different experimental conditions). After the display disappeared, a question about the display was presented word by word. **Bottom panel:** response to nouns at which either the adjective had already resolved reference, or where the noun itself resolved reference. Plots are analogous to the top panel. Topographies are contrasted to the response to the nouns at which reference had not been resolved yet.

With the intention of establishing a neural marker of successful reference resolution for simple, unambiguous referential expressions, we sought to take advantage of the simplicity of the visual world paradigm. In its canonical form the visual world paradigm is not well suited for EEG data collection, where eye movements cause artifacts that overshadow brain signals. In order to overcome this problem, we modified the mode of presentation: In each trial, the visual world display was only shown for a short time and then replaced by a question presented centrally, word by word (see illustration in Figure 1). Participants’ task was to focus on answering the question, using an internalized representation of the display hypothesized to reside in visual short term memory.

This paradigm allowed us to capitalize on a well-known effect from the literature on visual short-term memory: Directing attention to one side of the visual field is associated with a negative evoked potential at posterior electrodes contralateral to the side of attention (henceforth: “posterior contralateral negativity”). Originally described as an attention-dependent enhancement of the stimulus-dependent N2 component (e.g., Eason et al., 1969; Vanvoorhis and Hillyard, 1977; Heinze et al., 1990) this effect also occurs as a sustained posterior contralateral negativity when participants are instructed to maintain stimuli from only one side of a display in short term memory (e.g., Klaver et al., 1999; Vogel and Machizawa, 2004). Interestingly, a posterior contralateral negativity can also be observed in response to a centrally presented stimulus if the task requires relating it to an earlier, lateralized presentation (Gratton et al., 1997; Kuo et al., 2009; Dell’Acqua et al., 2010; Eimer and Kiss, 2010). These studies suggest that visual short-term memory traces are encoded and accessed in a topographic manner related to modality-specific neural pathways (cf. Klaver et al., 1999). Based on these results, we hypothesized that reference resolution in a referential domain held in visual short-term memory similarly entails access to modality-specific memory traces, which should manifest in a posterior contralateral negativity.

Some evidence concerning the involvement of the visual system in language processing comes from visual world studies in which the visual world display was shown only initially, and then removed and followed by a blank screen while participants listened to a sentence referring to objects in the display. Even on that blank screen, participants tended to fixate the previous location of the mentioned objects (Altmann, 2004; Altmann and Kamide, 2009). This was interpreted as reflecting access to an internal scene or event representation linking objects with their (prior) spatial location, and suggests that even in the absence of a concurrent visual display reference resolution in visual scenes held in short-term memory involves at some level access to a modality-specific visuo-spatial representation.

## Materials and Methods

### Participants

We collected EEG data from 14 native speakers of English at New York University, Abu Dhabi. Data collection happened on a subset of participants taking part in a larger magnetoencephalography (MEG) study testing a different set of hypotheses. All participants had normal or corrected-to-normal vision, and none were colorblind or had known neurological abnormalities. Data from one subject were excluded because fewer than 50% of the trials remained after artifact rejection, leaving eight female and five male participants in the final analysis (mean age 24.3, range 18–38 years). All participant had acquired English as their first language, but three of the 13 grew up speaking at least one other language. The protocol was approved by the Institutional Review Board of NYU Abu Dhabi, and all participants provided written consent before beginning the experiment.

### Design and Stimuli

Each trial consisted of a visual world display and a corresponding question (see Figure 1, middle panel for an illustration). The visual world display and content words were presented for 300 ms with an interstimulus interval (ISI) of 300 ms, whereas short function words like “was,” “the,” “on,” and “in” were presented for 200 ms with an ISI of 200 ms. The last word of the question stayed on the screen until participants gave a yes/no answer by pressing one of two buttons.

Each visual world display contained three horizontally aligned objects. Objects were constructed on the basis of six colors (blue, brown, green, pink, red, white) and five shapes (boat, fish, heart, star, truck). There were two kinds of displays (a manipulation that was mainly of interest for the MEG study and is not discussed further here): The first, simpler kind contained one pair of objects that shared color and another pair that shared shapes, with all shapes visible. The second, more complex kind of display contained one object of unique color, but with its shape occluded, and two additional objects, both of the same color and shape, but with differing patterns.

All questions began with “Was the [color-adjective] [shape-noun] …”, and asked about the absolute or relative position of one of the items in the display, for example “Was the pink fish next to a boat?” or “Was the blue heart in the middle?”. Different kinds of questions were used to discourage participants from relying on specific strategies focusing on particular aspects of the visual displays. The correct answer was “yes” in half of the trials and “no” in the other half. The color adjectives and shape identifying nouns used in referential expressions (*blue*, *brown*, *green*, *pink*, *red*, *white*; *boat*, *fish*, *heart*, *star*, *truck*) were all common words with SUBTL frequencies between 28.5 and 244.2 per million words (Brysbaert and New, 2009) and mean lexical decision latencies between 523 and 653 ms according to the English Lexicon Project (Balota et al., 2007).

The complete design included the factors reference (adjective resolving vs. adjective non-resolving), display kind (simple or complex), location of the referent (left, middle, right, with middle treated as fillers for the purpose of this analysis), color (six levels) and shape (five levels) for a total of 2 × 2 × 3 × 6 × 5 = 360 trials. Each participant saw the same 360 trials, but the order was randomized for each experimental session. Thus, for each possible location of the referent (left, middle, right), there were 60 trials in which reference was resolved by the adjective (top left and top right displays in Figure 1). In 30 trials the noun resolved reference (bottom left display in Figure 1), and in another 30 trials reference was resolved by a prepositional phrase following the noun (bottom right display in Figure 1).

### Procedure

Participants were given instructions on the reference task and allowed to practice using sample trials until they felt comfortable performing the task. Recordings took place concurrent with MEG recordings, inside a magnetically shielded MEG acquisition chamber. Participants lay in a supine position and stimuli were projected onto a horizontal screen at comfortable viewing distance. Participants were instructed to blink as little as possible during the presentation of the stimuli. They were told that if they needed a break they could withhold their yes/no response at the end of a trial until they felt comfortable to continue. In regular intervals throughout the experiment they were informed of the progress in the experiment with a text display and had the opportunity to take a short, self-terminated break. Stimuli were presented with MATLAB using psychtoolbox^1^ and ptbwrapper^2^. On average, an experimental session took 42 min from first to last trial (without setup).

Data were recorded from 31 EEG and 3 EOG electrodes attached to an elastic cap at standard positions in the international 10–20 system (EasyCap GmbH, Germany) at a sampling rate of 1000 Hz. Impedances were kept below 10 kΩ. The ground was located at the AFZ electrode position, and recordings were referenced to the left mastoid electrode. The signal was amplified with a BrainVision Brain Amp Standard amplifier.

### Data Analysis

Data were pre-processed and analyzed with MNE-Python (Gramfort et al., 2013, 2014) and Eelbrain^3^. Raw data were band-pass filtered offline between 0.1 and 40 Hz. We extracted epochs from –100 to 600 ms relative to the onset of the adjectives. Epochs containing artifacts were excluded from further analysis, and individual channels containing noise were interpolated. Artifact rejection proceeded with automatic rejection of epochs with a signal exceeding an absolute 7.5 μV threshold, followed by adjustment based on visual inspection. If individual channels exhibited signal at abnormal amplitude independently of neighboring channels, the signal at aberrant channels was interpolated using spherical spline interpolation from the remaining channels instead of rejecting the whole epoch. Epochs were baseline corrected using the 100 ms pre-adjective period and re-referenced to the average of the two mastoid electrodes.

The statistical analysis focused on the lateral posterior electrodes, O1, O2, P3, P4, P7, and P8. These electrodes represent the lateral posterior part of the head in our electrode layout, where N2pc and contralateral delay activity are most reliably observed (see literature cited in the introduction). For each subject and electrode pair (O1/O2, P3/P4, and P7/P8) we computed one wave form for adjectives resolving reference to the side contralateral to the electrode, and a second waveform for adjectives resolving reference to the side ipsilateral with the electrode. We then tested the hypothesis that the contralateral signal was more negative than the ipsilateral signal with temporal cluster-permutation tests based on one-tailed *t*-tests (see Nichols and Holmes, 2002; Maris and Oostenveld, 2007). We performed the test on a time window from 200 to 500 ms after adjective onset. The beginning of this time window was based on the onset of the posterior contralateral negativity in visual short-term memory studies around 200–250 ms (e.g., Dell’Acqua et al., 2010) and the offset was based on when people moved their eyes to the referent identified by an adjective in visual world studies (Eberhard et al., 1995). For each time point (at a resolution of 1000 Hz) we calculated a related-samples *t*-value. We then formed clusters based on contiguous regions of *t*-values greater or equal to a value equivalent with an uncorrected one-tailed *p*-value of 0.05. For each cluster we calculated the cluster mass (i.e., the sum of the *t*-values making up the cluster). We then repeated this procedure with all 8191 possible permutations of the data (with 13 participants there are 2^13^–1 possible ways of switching condition labels within subjects) and extracted for each permutation the maximum cluster mass value. The distribution of these values provides a non-parametric estimate of the expected distribution of the maximum cluster mass statistic under the null hypothesis. This distribution was used to assign to each cluster in the actual data a *p*-value corrected for multiple comparisons across time, by locating the cluster’s mass on the distribution. Since we performed this procedure at three electrode pairs we multiplied all resulting *p*-values by 3.

We analyzed the response to nouns with the same procedure with epochs extracted around the onset of presentation of the nouns. Due to the low number of trials in which the noun actually resolved reference, we performed a cluster-permutation analysis over trials in which reference was resolved by the adjective or the noun, and used the time window identified by this analysis for targeted *post hoc* tests in the sub-conditions.

## Results

### Behavioral

On average, participants answered 87.6% of the questions correctly, ranging from 77.2 to 97.8% correct.

### EEG Response to Adjectives

After artifact rejection, an average of 55.2 trials per participant per condition (reference resolution to the left, reference resolution to the right) remained of a total of 60 possible. A significant cluster in which the signal contralateral to the referent was more negative than the ipsilateral signal was found in the O1/O2 sensor pair (333–379 ms, *p* = 0.019 Bonferroni-corrected). Figure 1 (top panel, left side) shows the contralateral and ipsilateral response to the adjectives, with the time window of the significant cluster shaded. The accompanying topographic plots show the average potential, during the time window identified by the cluster, for reference resolution toward the object on the left (or the right) side of the display minus the average of the non-resolving adjectives, illustrating the presence of the posterior contralateral negativity.

### EEG Response to Nouns

Our initial analysis of the response to the noun included all trials in which after reading the noun, participants could know the location of the referent. This included trials in which reference was resolved by the adjective and trials in which reference was resolved by the noun. This yielded an average of 82.0 trials per referent location condition (referent on the left vs. referent on the right) out of 90 possible. In this combined response, we found a significant posterior contralateral negativity at the P3/P4 electrode pair (395–454 ms, *p* = 0.025 Bonferroni-corrected) and at the P7/P8 electrode pair (383–449 ms, *p* = 0.027 Bonferronicorrected). Figure 1 (lower panel) shows the contralateral and ipsilateral responses at P3/P4 and P7/P8. To illustrate the topography of the effects, the figure shows topographic maps in which the response to nouns with known referents on the left or right side of the display is compared to the response to nouns at which the location of the referent was still unknown.

For follow-up analysis in the sub-conditions we calculated the average of the P3/P4 and P7/P8 sensor pairs in the time window 395–449 ms, in which the two clusters overlapped. This analysis confirmed the recurrence of a posterior contralateral negativity in the response to nouns in the adjective resolving condition [difference = –5.34 μV, *t*(12) = 3.50, *p*_one–tailed_ = 0.002]. In the noun-resolving condition, in which the nouns followed adjectives that were compatible with two objects, the difference was in the expected direction, but did not reach significance [difference = –2.98 μV, *t*(12) = 1.12, *p*_one–tailed_ = 0.14]. This result begs the question whether we simply lacked the power to detect the response to resolving nouns due to the low number of trials in this condition, or whether the response to resolving nouns was indeed different form the response to non-resolving nouns. This latter hypothesis would predict a significant difference between the contralateral negativity in the response to resolving and non-resolving nouns; however, a related measures *t*-test indicated that this was not the case [difference = 2.36 μV, *t*(12) = 0.72, *p* = 0.49]. Therefore, our data do not let us draw a conclusion about the response to reference-resolving nouns.

One possible explanation for a contralateral response to nouns after reference-resolving adjectives is that on some trials, readers failed to resolve reference on the adjective, even though this would have been possible, and caught up by resolving reference when they read the noun. This line of reasoning would suggest a negative relationship, trial by trial, between the contralateral negativity on the adjective and the contralateral negativity on the noun. In order to test whether this was the case we calculated, for each subject, within the adjective-resolving trials, the correlation coefficient between the contralateral response to the adjectives and the contralateral response to the nouns. The contralateral effects were quantified as the contralateral minus ipsilateral difference of the average of the time points and sensors involved in the significant clusters described above. A one sample *t*-test indicated that these correlation coefficients were not reliably different from 0 across subjects [mean *r* = –0.01, *t*(12) = –0.53, *p* = 0.61]. This indicates that the contralateral response to the nouns in cases where the adjective had already resolved reference was not contingent upon the absence of a contralateral response to the adjective, i.e., that participants tended to show a contralateral response to both words.

## Discussion

We investigated whether a posterior contralateral EEG response previously observed in visual short-term memory tasks is also present when linguistic expressions refer to objects held in visual short-term memory. We analyzed the response to visually presented adjective–noun phrases, embedded in a natural context of questions about visual displays. As predicted, we found that reference resolution was associated with a negative evoked potential at posterior electrodes contralateral to the site of the referent.

When adjectives resolved reference (i.e., color adjectives in contexts where only one object had that color) they were associated with a posterior negativity contralateral to the referent, starting 333 ms after presentation of the adjective. Importantly, the conditions we compared involved the same adjectives; what differed between conditions was the location of the referent picked out by the adjectives. The fact that the signal reflected the spatial position of the referent within the referential domain strongly implies that it was due to a process associated with reference resolution, for which that location mattered, rather than a process that is independent of the location of the referent (such as, for example, a cloze probability effect).

Our results leave open the exact nature of the process that produces the observed effect. On the one hand, the effect does not necessarily reflect commitment to one specific object as the referent for the given linguistic expression; it would also be compatible with an evaluation process supporting reference resolution, for example by activating those spatial locations in a visual short-term memory representation that include the color named by the adjective. On the other hand, an alternative possibility is that reference is resolved in an abstract, modalitygeneral representation, and the visual representation is only accessed once the referent is found in the abstract representation. These questions are open to future research. However, the presence of a an EEG signal that reflects the spatial position of the referent does suggest that contact between the semantics of the word (color adjective) and the referential domain has been established, and that the adjective leads readers to activate the portion of the referential domain that contained the item with the corresponding color.

The response to adjectives reflecting a reference resolution process is consistent with findings from visual world studies which showed that reference resolution is incremental, i.e., that language comprehenders use each incoming word of a referential expression to constrain the set of possible referents (Eberhard et al., 1995). In addition, while visual world studies used spoken language input, our results extend this observation to the context of reading.

For nouns, the posterior contralateral negativity started around 383 ms and was significant even when the noun merely occurred as the head of an expression for which reference had already been resolved. This indicates that even when readers had supposedly already identified the referent, they still reactivated the corresponding representation when processing the noun. This finding could be relevant for models of the comprehension of overspecified referential expressions (for an overview, see Gatt et al., 2014). Language producers frequently overspecify referential expressions, in particular involving colors, for example, using *the blue heart* in a context in which *the heart* would have been sufficient to distinguish the referent from its competitors (Pechmann, 1989). In simple contexts, overspecified expressions have been argued to speed up (e.g., Arts et al., 2011) or slow down comprehension (e.g., Engelhardt et al., 2011). Our results suggest that our participants processed redundant information by reactivating the referent they had already identified through the adjective. This might indicate that, regardless of processing speed, overspecification is associated with more robust comprehension. For example, participants might have reactivated the representation of the referent when reading the noun to check their initial interpretation after the adjective. This increase in robustness could be particularly relevant in real life referential domains, which are often more complex and less constrained than experimental stimuli. Indeed, it has been shown that overspecification can significantly simplify the referential search in certain more complex referential domains (Paraboni et al., 2007; Paraboni and van Deemter, 2013).

While the latency difference between adjectives and nouns is suggestive, it would seem premature to draw definite conclusions, especially since our design did not include enough trials on which the noun resolved reference.

Our results put the time point at which readers identify a referent’s location in response to a visually presented content word around 350 ms. Even though the effect we described is of a quite different nature, it converges with studies of referential ambiguity (e.g., Van Berkum et al., 1999) to place the time point at which linguistic input starts interacting with the referential domain between 300 and 400 ms.

More broadly, the observation of a posterior negativity contralateral to the referent of a linguistic expression suggests that people use the same or similar memory systems when understanding language as in non-linguistic visual short-term memory tasks. If this interpretation is correct, it suggests that people use domain-specific, non-linguistic representations when comprehending referential expressions. This observation fits well into the broader context of research suggesting that language comprehension engages domain-specific cognitive mechanisms to process linguistic meaning (e.g., Zwaan et al., 2002). Our results suggest that it is possible to track the mind’s eye looking at a visual memory when reading about it.

### Conflict of Interest Statement

The authors declare that the research was conducted in the absence of any commercial or financial relationships that could be construed as a potential conflict of interest.

## Footnotes

1 psychtoolbox.org

2 code.google.com/p/ptbwrapper

3 https://pythonhosted.org/eelbrain
